# Venous valves of the head and neck: a narrative review

**DOI:** 10.1007/s00276-026-03910-1

**Published:** 2026-05-26

**Authors:** Mugurel Constantin Rusu, Diana Alexandra Bănică, Răzvan Costin Tudose

**Affiliations:** Division of Anatomy, Department 1, Faculty of Dentistry, Carol Davila University of Medicine and Pharmacy, 050474 Bucharest, Romania

**Keywords:** Venous valves, Head and neck, Internal jugular vein, Facial vein, Cavernous sinus, Microvascular surgery, Cerebral venous hemodynamics, Scale for the assessment of narrative review articles

## Abstract

**Background:**

Classical anatomical teaching has long described head and neck veins as largely valveless, allowing free bidirectional flow. Modern cadaveric, imaging, and surgical studies challenge this oversimplification.

**Objective:**

This narrative review synthesises current anatomical evidence regarding the presence, morphology, distribution, and functional significance of venous valves throughout the head and neck venous system.

**Methods:**

A literature search was conducted across PubMed and Semantic Scholar. The review was structured following the Scale for the Assessment of Narrative Review Articles (SANRA) quality framework. All primary research references were verified against PubMed.

**Results:**

Valves have been documented in the facial, lingual, labial, pharyngeal, superior ophthalmic, superficial temporal, glabellar/forehead, external jugular, and internal jugular veins, as well as at the lymphovenous junction. These valves are predominantly bicuspid and cluster near venous junctions. The internal jugular vein has a valve near its termination in approximately 86–93% of specimens; functional competence is context-dependent, being present in patients with normal central venous pressure but frequently absent in those with chronically elevated pressure, and further compromised by cannulation trauma. Jugular venous reflux from valve insufficiency has been associated with several neurological conditions. The cerebral venous system, dural sinuses, emissary veins, and the internal vertebral venous plexus are consistently valveless.

**Conclusions:**

Contemporary evidence demonstrates that head and neck veins are not uniformly valveless. Valve presence and competence carry implications for infection spread, microvascular surgery, central venous cannulation, and cerebral hemodynamics.

## Introduction

Classical anatomy teaching often emphasises that much of the intracranial venous circulation lacks valves and that the cerebral venous system contains neither valves nor a muscular layer [33]. This traditional characterisation has been extrapolated in many teaching resources to suggest that the entire head and neck venous territory is valveless, with the corollary that blood can flow freely in either direction, a concept central to the classical explanation of how facial infections spread to the cavernous sinus.

The controversy over valves in head and neck veins spans four centuries of anatomical scholarship. Fabricius ab Aquapendente (1537–1619) illustrated valves at the entry of the internal jugular vein in his De venarum ostiolis (1603), but declared that the remainder of the IJV, the superior vena cava, and most cutaneous veins were valveless. His pupil William Harvey, who studied at Padua from 1600 to 1602, grasped the true significance of venous valves for unidirectional circulation. Franklin’s 1927 survey of four centuries of literature codified the terminology still employed today – ‘ostial’ valves at tributary orifices and ‘parietal’ valves arising from the vein wall, with the bicuspid form the most common in man – and noted that while Haller had recorded valves in the facial, lingual, and tonsillar extracranial branches of the IJV, only occasional valves in the angular and ophthalmic veins were conceded by Piersol [19]. This conservative characterisation, embedded in generations of anatomical textbooks, is now substantially revised by modern cadaveric, scanning electron microscopy, and imaging evidence [10].

In contrast, modern anatomical and clinical literature clearly document valves in major cervical outflow pathways, such as the IJV and the external jugular vein (EJV), with significant inter-individual variability and clinically relevant patterns of competence and incompetence [2, 61, 66]. Cadaveric dissection studies using corrosion resin casts and stereomicroscopy have identified numerous bicuspid valves throughout the facial venous system [44, 47, 70]. Valves have also been demonstrated in the superior ophthalmic vein (SOV) [28, 70], the superficial temporal vein (STV) [67], the glabellar and forehead venous networks [57], and the lymphovenous junction of the thoracic duct [51].

**Fig. 1:**
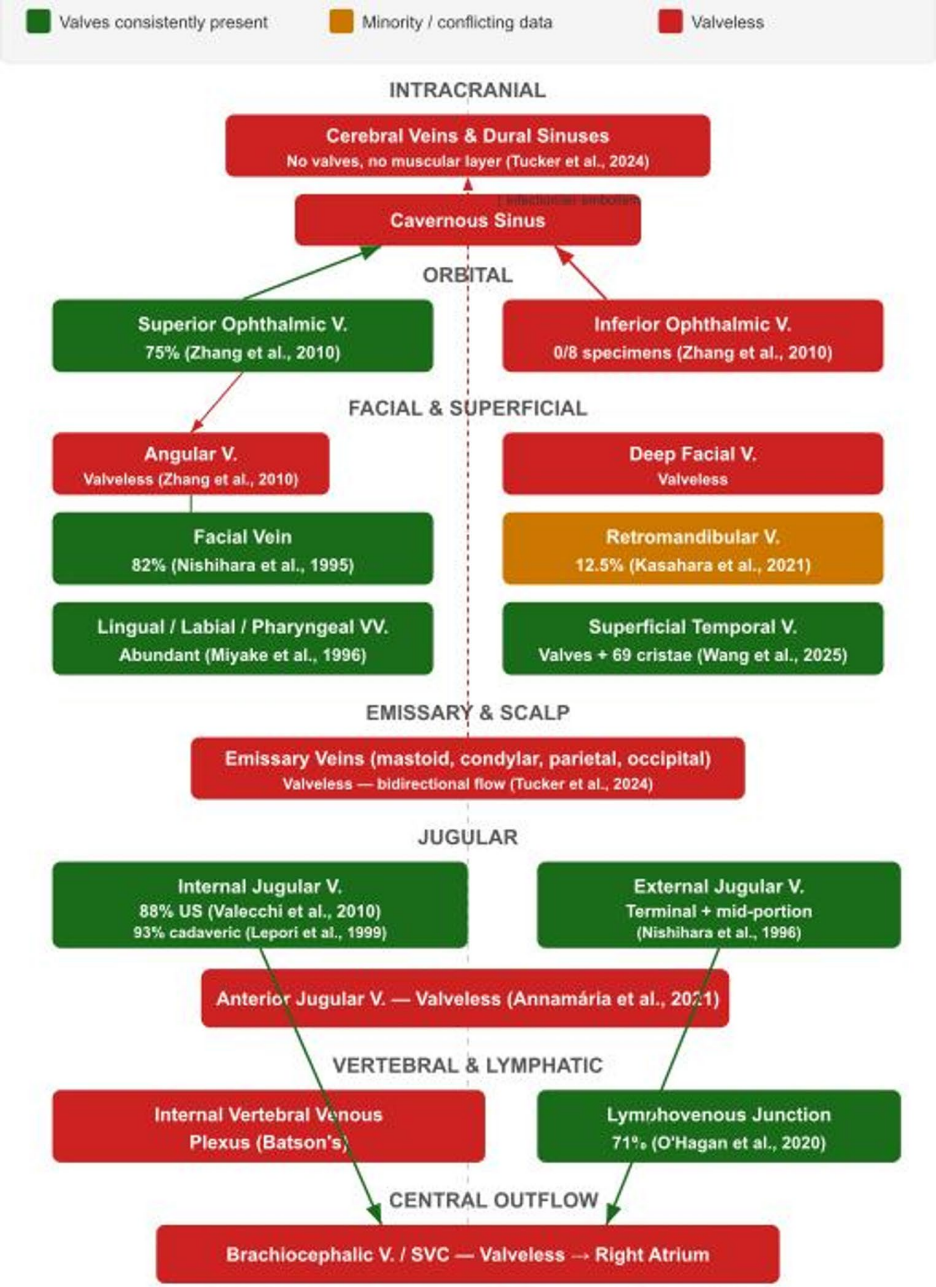
Valve distribution map of the head and neck venous system. Schematic diagram organised by anatomical zone (intracranial, orbital, facial, emissary, jugular, vertebral, central outflow). Venous structures are colour-coded by valve status: green = valves consistently present (IJV, EJV, facial vein, SOV, STV, lingual/labial/pharyngeal veins, lymphovenous junction); orange = valves present in minority or conflicting data (retromandibular vein, angular vein); red = valveless (cerebral veins, dural sinuses, emissary veins, IOV, AJV, IVVP, brachiocephalic vein/SVC). Prevalence percentages are annotated where available. Connecting arrows indicate principal flow directions

**Fig. 2:**
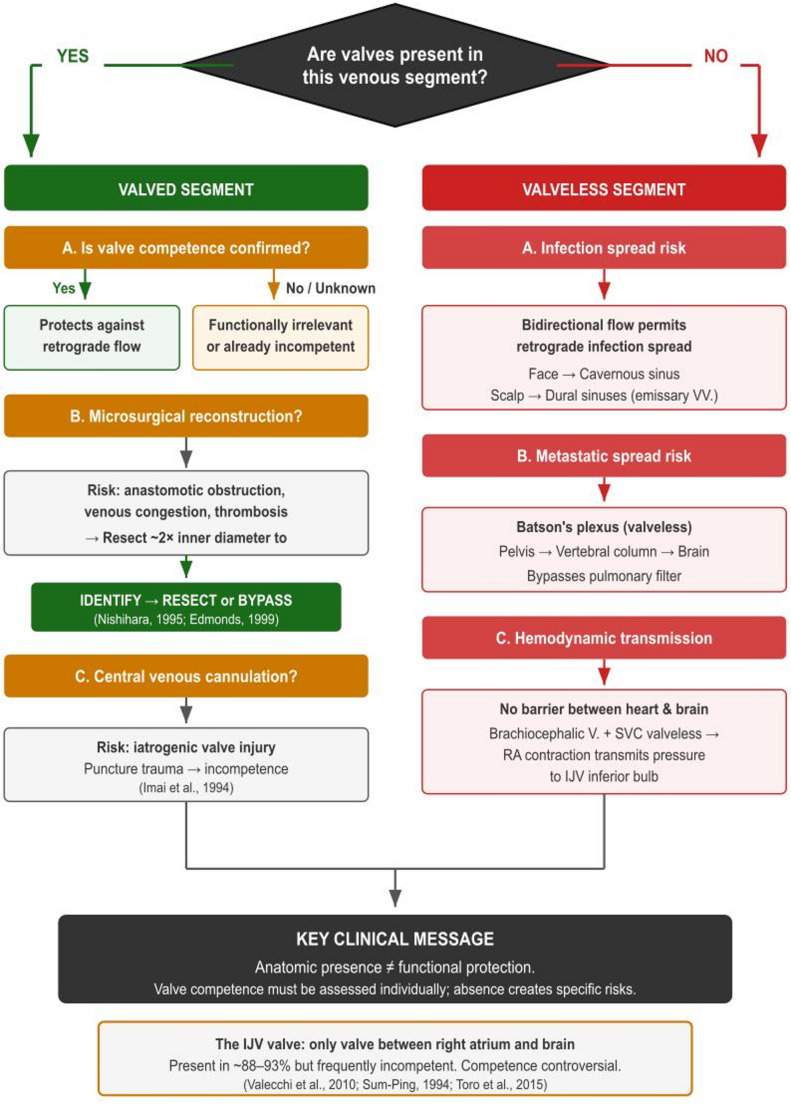
Clinical implications of head and neck venous valve status. Decision flowchart originating from the central question “Are valves present in this venous segment?” Two arms branch from this question. The YES (valved) arm addresses three clinical domains: (A) competence assessment (competent valves protect against retrograde flow; incompetent valves offer no functional protection), (B) microsurgical reconstruction (risk of anastomotic obstruction; management by identifying or resecting approximately twice the inner diameter), and (C) central venous cannulation (risk of iatrogenic valve injury through puncture trauma). The NO (valveless) arm addresses: (A) infection spread risk (bidirectional flow permitting retrograde infection via face–cavernous sinus and scalp–dural sinus pathways), (B) metastatic spread risk (IVVP/Batson’s plexus bypassing pulmonary filter), and (C) hemodynamic transmission (absence of barrier allows right atrial pressure transmission to cerebral circulation). Both arms converge on the key message that anatomic presence does not guarantee functional protection. An inset box highlights the IJV valve as the only valve between the right atrium and the brain, noting its prevalence and the controversy surrounding its competence

**Fig. 3:**
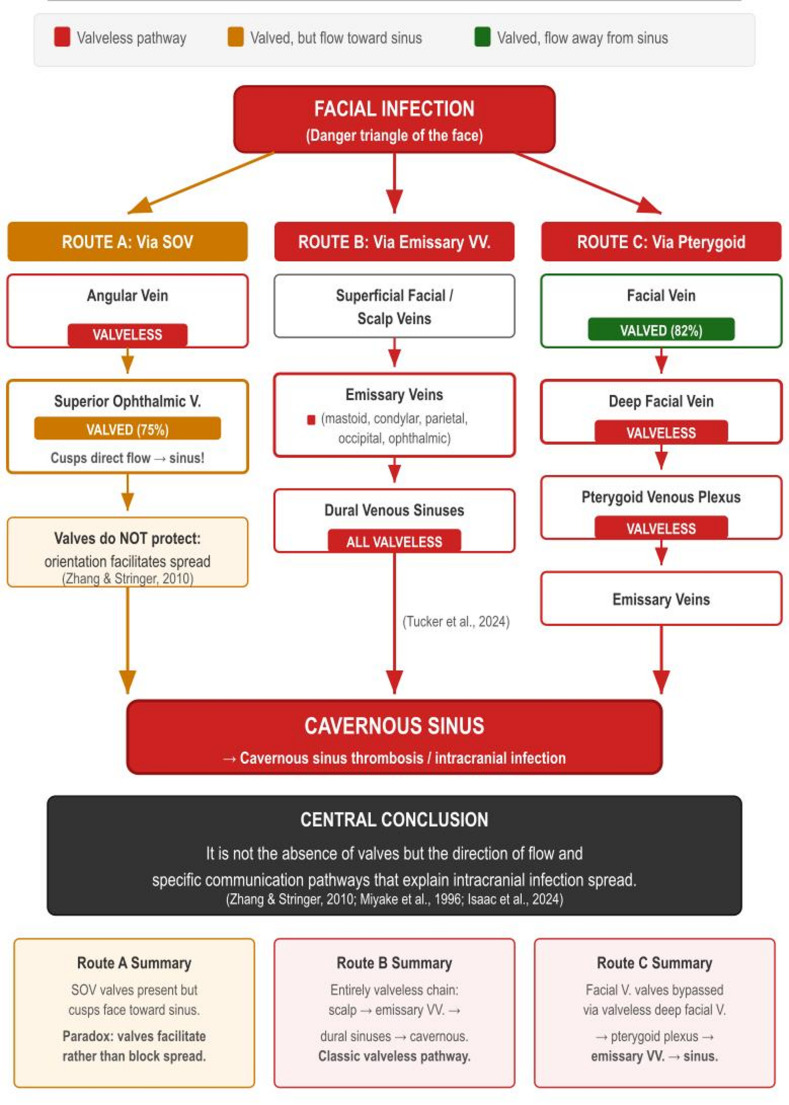
Infection spreads from the face to the cavernous sinus. Flow diagram tracing three parallel routes from a facial infection (danger triangle) to the cavernous sinus. Route A (via SOV): angular vein (valveless) → SOV (valves present in 75%, but cusps direct flow *toward* cavernous sinus) → cavernous sinus. Paradoxically, valves facilitate rather than block spread [70]. Route B (via emissary veins): superficial facial/scalp veins → emissary veins (valveless) → dural sinuses (valveless) → cavernous sinus. The classic entirely valveless chain [33]. Route C (via pterygoid plexus): facial vein (valved, 82%) → deep facial vein (valveless) → pterygoid venous plexus (valveless) → emissary veins → cavernous sinus. Facial vein valves are bypassed via the valveless deep facial vein [52, 59]. Central conclusion: It is not the absence of valves but the direction of flow and specific communication pathways that explain intracranial infection spread

This narrative review aims to synthesise the contemporary anatomical evidence regarding the presence, morphology, distribution, and functional significance of venous valves throughout the head and neck venous system (Fig. 1), and to discuss the clinical implications for surgical practice, venous access, cerebral hemodynamics (Fig. 2), and infection spread (Fig. 3).

## Methods

This narrative review was conducted and structured in accordance with the Scale for the Assessment of Narrative Review Articles (SANRA), a validated quality assessment tool for non-systematic reviews [5]. SANRA comprises six items rated from 0 (low standard) to 2 (high standard): (1) justification of the review’s importance, (2) statement of aims, (3) description of the literature search, (4) referencing, (5) scientific reasoning and presentation of evidence level, and (6) presentation of relevant endpoint data. Each domain was addressed as follows.

### Justification and aims (SANRA Items 1–2)

The significance of revisiting the venous valve anatomy of the head and neck is justified by the ongoing discrepancy between traditional teaching (a valveless system) and the growing anatomical evidence (valves found in multiple areas). The specific aim – to synthesise valve presence, morphology, distribution, and clinical relevance across all head and neck venous regions – was outlined in the Introduction.

### Literature search (SANRA Item 3)

A structured literature search was conducted in PubMed using the following terms: “venous valves AND (head OR neck OR jugular OR facial OR ophthalmic OR temporal OR emissary OR vertebral)”. Additional searches were performed in Semantic Scholar and Consensus (an academic search engine) to find non-PubMed-indexed anatomical literature. Reference lists of included articles were hand-searched. No date restrictions were applied. The search was last updated in March 2026.

Search records were screened in three stages: first, title and abstract review for relevance to venous valves or valveless venous pathways in the head and neck; second, full-text review for direct anatomical, imaging, surgical, or physiological evidence; and third, regional mapping into jugular, facial/superficial, ophthalmic, intracranial-emissary, vertebral, and lymphovenous categories. Data were extracted narratively for valve presence, morphology, location, competence, flow direction, and clinical implications; no quantitative meta-analysis was attempted because of heterogeneity in study design, specimen type, and outcome reporting.

**Fig. 4:**
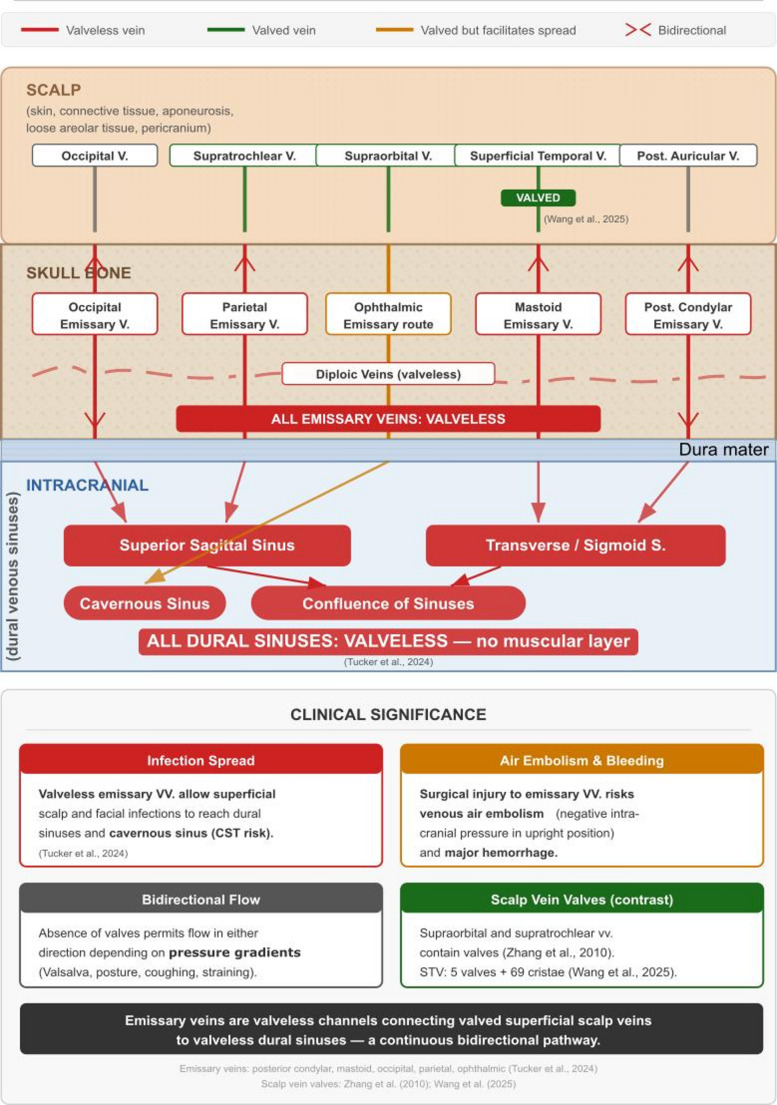
Veins of the scalp and emissary veins: layered anatomical schematic. Cross-sectional diagram showing the relationship between superficial scalp veins, emissary veins traversing the skull, diploic veins within the diploe, and intracranial dural venous sinuses. The diagram is organised into four anatomical layers, from superficial to deep: the scalp, the skull bone, the dura mater, and the intracranial compartment. Five named scalp veins are shown: occipital, supratrochlear, supraorbital, superficial temporal, and posterior auricular. Of these, the supraorbital and supratrochlear veins contain valves [70], and the superficial temporal vein harbours both bicuspid valves and valve-like cristae [67]. Four named emissary veins are shown traversing the skull: occipital, parietal, mastoid, and posterior condylar; the ophthalmic venous route is depicted separately via the superior ophthalmic vein/cavernous sinus pathway. All named emissary veins are valveless, permitting bidirectional flow indicated by paired chevron arrows [33]. Diploic veins (dashed red line) run within the diploe and are likewise valveless. Intracranially, the superior sagittal sinus, transverse/sigmoid sinus, confluence of sinuses, and cavernous sinus are shown; all are valveless and lack a muscular layer [33]. Arrows indicate drainage directions from emissary veins into dural sinuses; the ophthalmic venous route drains toward the cavernous sinus (orange arrow). The lower panel summarises four clinical implications: infection spreading to the cavernous sinus; the risk of venous air embolism and haemorrhage from surgical injury; bidirectional flow governed by pressure gradients; and the contrasting presence of valves in scalp veins. Colour coding: green = valved, red = valveless, orange = valved but facilitating spread toward the cavernous sinus

The review structure follows the regional valve map in Fig. 1; clinical consequences are organised in Fig. 2, infection pathways in Fig. 3, and the scalp-emissary venous relationships in Fig. 4.

### Referencing (SANRA Item 4)

All primary research references were individually verified against PubMed (with PMID confirmation where available). Non-peer-reviewed web sources identified in preliminary searches (educational websites, slide-sharing platforms) were excluded and replaced with indexed, peer-reviewed publications.

### Evidence level and endpoint data (SANRA Items 5–6)

The level of evidence for each anatomical claim is indicated in the text (e.g., cadaveric dissection, ultrasound imaging, autopsy series, corrosion resin casts). Where available, quantitative prevalence data with sample sizes are reported (Table 2). Conflicting findings between studies are explicitly noted and discussed.

## Jugular veins

### Internal jugular vein

#### Anatomical presence and morphology

Multiple studies report that valves are commonly present in the distal IJV. A cadaveric study found distal IJV valves in 93% of specimens [37], while ultrasonographic evaluation of 462 IJVs demonstrated valve leaflets in approximately 88%, all located in the distal segment [66]. However, valve presence is variable; reports indicate anatomic absence in approximately 12% of cases, and some autopsy series have documented specimens with no valves, including in left IJVs [22, 42].

When present, IJV valves are most often bicuspid, although unicuspid and tricuspid morphologies have also been described. A cadaveric study of 97 specimens found bicuspid valves in 66%, unicuspid valves in 15%, tricuspid valves in 6%, and no valves in 13% of IJVs [2]. In a large in vivo ultrasound series of 240 IJVs in 120 healthy subjects aged 18–90 years, a valvular apparatus was identified in 86% of cases, predominantly bicuspid (75%), with unicuspid (15%) and tricuspid (10%) morphologies also observed [38]. In another autopsy cohort of 100 cadavers, the right IJV harboured bicuspid valves in 47% and the left tricuspid valves in 62%, illustrating consistent right–left asymmetry [56]. Anatomically, IJV valves are consistently reported near the lower IJV, including around the ostium/termination region. One series placed them approximately 1.2 cm (right) and 1.8 cm (left) above the brachiocephalic junction [56], and another approximately 28 mm from the IJV termination on ultrasound [38].

#### Functional competence

Anatomic presence should not be equated with functional competence. Functional competence remains a central point of controversy, with findings varying substantially across methodologies and study populations. An early landmark study using catheter-measured transvalvular pressure gradients and venography demonstrated competent right IJV valves in all 17 patients with normal central venous pressure, with peak transvalvular gradients of 52.4 ± 8.6 mmHg during cough; in contrast, 10 of 15 patients with chronically elevated central venous pressure had incompetent or absent valves, with absent valves found exclusively in cases of long-standing severe tricuspid regurgitation [18]. The same study confirmed competent valves in the left IJV and bilateral subclavian veins under equivalent pressure challenges [18]. In vivo ultrasound under standardised insufflation revealed incompetence in 95% of IJV valves across a broad adult age range [38]. A larger colour Doppler series of 462 IJVs found incompetence in 90% of subjects, with competent valves observed only in individuals under 20 years of age [66]. These ultrasound findings contrast with cadaveric and in vivo pressure studies: Silva et al. (2002) found the IJVV present in all 60 IJVs from 30 cadavers, competent up to a median maximum hydrostatic pressure of 56.8 mmHg (range 32.2–96 mmHg), and competent in all 25 live subjects studied by colour duplex scanning during a 60 mmHg Valsalva manoeuvre – no reflux was detected in any live subject [58]. A paediatric ultrasound series of 120 children and adolescents aged 3–20 years found IJV valves in virtually all subjects, with competence demonstrably higher in younger age groups and declining significantly in older subjects, suggesting progressive age-related deterioration of valve function that may explain the high incompetence rates reported in adult series [13]. Cannulation of the IJV is a further recognised cause of iatrogenic valve damage: a prospective Doppler ultrasound study in 91 surgical patients demonstrated that proximal cannulation induced valve incompetence in 76% of cases versus 41% for distal cannulation, and this incompetence persisted at follow-up 8–27 months after catheter removal in most affected patients [68]. These data indicate that choosing a more distal cannulation site may reduce, but does not eliminate, the risk of permanent valve injury [27, 68].

Clinically, the IJV valve has been characterised as the sole valve between the right atrium and the brain [27, 64]. Its competence is critical for maintaining the extrathoracic arteriovenous pressure gradient required for forward blood flow during cardiopulmonary resuscitation and for protecting the cerebral circulation from acute increases in intrathoracic pressure during coughing or positive-pressure ventilation [18, 56]. Valve obstruction has been reported to cause venous reflux and intracranial venous hypertension [17, 64]. Jugular venous reflux (JVR) resulting from valve insufficiency – whereby retrograde jugular venous flow transmits central venous pressure to the cerebral circulation – has been significantly associated with a range of neurological conditions, including transient global amnesia, transient monocular blindness, multiple sclerosis, exertional headache, and idiopathic intracranial hypertension; however, the causal status of JVR in these disorders remains unestablished, and further investigation is needed [26]. IJV valves are also relevant during CPR and jugular vein cannulation [27, 56]. Systematic reviews have further highlighted the variability in valve anatomy and its clinical complications [35, 49]. The overall valve distribution across the head and neck is summarised in Fig. 1.

Histologically, the IJV valve leaflets (valvulae venosae) are evaginations of the tunica intima and tunica media. Each leaflet consists of an endothelial fold stiffened by a dense collagen fibre network, with no muscle cells or blood vessels within the leaflet itself [56]. Age-related changes include a progressive increase in collagen and elastin content of the intima and media, wall thickening with irregular collagen bundles under chronic venous congestion, and gradual leaflet atrophy leading to physiological insufficiency in later decades. In patients with established cardiac insufficiency, only rudimentary valve remnants may persist [56]. The subclavian vein, which is in direct continuity with the IJV, also consistently harbours valves, being bicuspid in 75% of cases, unicuspid in 12%, tricuspid in 4%, and absent in 9%; unlike the IJV, subclavian valves are distributed along the entire length of the vessel rather than confined to a single ostial site [2]. The brachiocephalic veins are generally valveless, though rare bicuspid substitute valves have been described, particularly in the right brachiocephalic vein, where they appear to functionally replace absent IJV bulb valves [2].

### External jugular vein

The EJV is consistently described as having valves at its terminal end before entering the subclavian vein, which inhibit regurgitation from the subclavian [33]. Dedicated anatomical studies show that EJV valves extend into the middle portion, a region used for microvascular anastomosis; bicuspid valves are common, though small or rudimentary [48]. Clinically, EJV valves affect microvascular anastomosis and increase the risk of thrombogenesis; resecting approximately twice the inner diameter may avoid the valve segment [48]. The EJV’s variable course and valves also make it unreliable for assessing jugular venous pressure [33].

Importantly, the EJV exhibits extensive morphological variability that must be considered alongside its valve anatomy. A comprehensive pictorial review documented fenestrations, true and false duplications, triplication, complete absence (reported in up to 14.2% of cases), aberrant origin or course, and rare drainage into the IJV rather than the subclavian vein [55]. The mean EJV diameter is approximately 9.3 mm, and it shows an inverse correlation with IJV diameter. These anatomical variations are directly relevant to EJV cannulation, identification of the greater auricular nerve during surgery, and flap design in head and neck reconstruction. The extensive variability of EJV morphology underscores the importance of preoperative imaging when surgical procedures involve this vessel, particularly because unrecognised fenestrations or duplications may harbour additional valve sites that complicate microvascular anastomosis [55].

Rare persistent jugulocephalic venous communications between the cephalic vein and EJV may also contain bicuspid valves directing flow from the cephalic vein toward the EJV, adding another variant valve-bearing pathway relevant to cephalic/EJV catheterisation and neck surgery [69].

A dedicated cadaveric dissection study of 100 EJVs in 50 adult cadavers found EJV valves in an ostial or paraostial position in 98% of specimens; the valvular system was most often bi-valvular, consistently oriented to prevent retrograde blood flow from the heart towards the head, and competent in all examined veins, a finding that explains the well-known difficulty of retrograde injection beyond the EJV termination [14]. A small minority of EJVs harboured a single membranous cusp, interpreted as a poorly formed or incomplete valve and likely incontinent. Three termination types were characterised: Type I (60%) into the jugulo-subclavian venous confluence, Type II (36%) into the subclavian vein at a distance from the internal jugular junction, and Type III (4%) directly into the trunk of the IJV; the junction angle varied between 20° and 70°, with the smallest angles (and thus the greatest catheterisation difficulty) in Type II terminations [14]. These findings have direct implications for central venous catheterisation via the EJV, as the sharp angulation in Type II terminations may cause catheter misdirection or vessel perforation at the point of inflexion.

A comparable study of 50 newborn cadavers reported that EJV valves were most often bi-valvular, paraostial, and membranous, with the same three termination types identified: Type I predominated (72%), Type II in 26%, and Type III in only 2% of cases [34]. In contrast to adult findings, retrograde injection beyond the neonatal EJV valves was not markedly impeded, suggesting that the membranous character of neonatal cusps confers less mechanical resistance than the well-formed adult valves. The junction angle ranged from 25° to 60°, consistent with adult values [34]. The available literature characterises catheter passage and mechanical resistance rather than direct flow metrics across the measured junction angles; no study was identified that quantitatively relates venous flow or drainage to discrete angle values. Functionally, smaller junction angles, especially in Type II terminations, appear to impede catheter advancement and may redirect the catheter against the subclavian wall, whereas Type I terminations provide a more direct path into the central venous system. Awareness of these termination variants and angulations is important when inserting central venous catheters in neonates via the EJV, as catheter passage is relatively straightforward in Type I but may cause subclavian perforation in Type II.

From a clinical anatomical perspective, the EJV has been described as having two distinct pairs of valves: an inferior pair at its entry into the subclavian vein and a superior pair approximately four centimetres above the clavicle. The intervening segment is often dilated and has been termed the “sinus” of the EJV [63]. This two-tier arrangement aligns with the ostial and paraostial valve locations systematically described by Deslaugiers et al. (1994) and corroborated by Kopuz and Akan (1996). The EJV’s outlet at the confluence of Pirogoff provides the anatomical basis for accessing the central venous territory via superficial puncture, provided the valve geometry and termination angle are taken into account [63]. A case series of 70 consecutive ICU patients reported a 90% success rate for central venous catheterisation via the EJV when the vein was macroscopically visible, with subcutaneous haematoma as the sole early mechanical complication in 7.93% of cases and no pulmonary complications [63].

The relative performance of EJV valves compared with IJV valves was recognised as early as 1880 by Gibson, who observed that valves at the proximal end of the EJV are less effective than those guarding the IJV for two physiological reasons: the EJV lies less directly in the line of the venous blood flow and therefore requires less robust valvular support; and the IJV valve, by contrast, is the critical barrier protecting the brain from venous congestion during acute rises in intrathoracic pressure [21]. This historical observation aligns with the modern finding that EJV valves, while consistently present in the ostial or paraostial position, are more susceptible to displacement or incompetence than their IJV counterparts.

Anatomical variants of the EJV have direct implications for interpreting its valve anatomy and for surgical planning. A case report of radical neck dissection for supraglottic carcinoma documented an anastomotic channel between the EJV and the IJV at the level of the thyroid cartilage, passing through the sternocleidomastoid muscle [31]. The anastomosis was located in the mid-portion of both veins, between a pair of valves situated approximately 2.5 cm above the vessel termination, and was fully functional without haemodynamic consequences. This case illustrates that the EJV valve anatomy must be interpreted in conjunction with potential inter-jugular communications, which may redirect venous flow between the superficial and deep cervical venous systems and create additional bypass routes relevant to surgical and catheterisation planning [31].

### Anterior jugular vein

The anterior jugular vein (AJV) is described as valveless [3]. Superficial infectious material may reach the cavernous sinus via connected venous pathways (Fig. 3).

## Facial and superficial veins

While some educational resources continue to describe facial veins as valveless, anatomic studies employing corrosion resin casts and stereomicroscopy have consistently documented valves [44, 47, 70].

### Facial vein

Valves were observed in 82% of facial veins, most frequently around the lower mandibular border [45, 47]. Bicuspid valves accounted for 93.5%; no tricuspid valves were observed [47]. Molinari et al. (2000) distinguished ostial (uni- or bicuspid) and parietal (bicuspid) valves [45]. Corrosion resin cast studies with scanning electron microscopy (SEM) revealed abundant valves in the lingual, labial, facial, and pharyngeal veins, particularly well-developed in the motile maxillofacial regions [44]. Crucially, SEM showed that valve leaflets in facial veins are extremely thin (∼4–6 μm) and that valves are present even in veins measuring less than 150–200 μm in diameter, below the threshold of conventional light microscopy and macroscopic dissection, explaining why prior studies reported their absence [44]. All facial vein valves encountered were bicuspid, and no valves were found in emissary veins [44]. Valve location and orientation directed flow from peripheral tributaries towards the facial vein and towards the IJV, consistent with a ‘muscle pump’ function in motile regions such as the lips, tongue, pharynx, and cheek [44]. Zhang and Stringer (2010) found 17 bicuspid valves in facial vein tributaries; cusp orientation predicted inferiorly directed blood flow. Surgical authors recommend avoiding valve-bearing segments at anastomosis sites [15, 47].

### Angular vein and muscular flow control

While conventional anatomical valves have not been consistently documented in the angular vein itself, a cadaveric study of 44 specimens revealed that the angular vein courses intimately through or between three facial muscles – the depressor supercilii (DS), orbicularis oculi (OOc), and zygomaticus minor (Zmi) – at specific, anatomically consistent points that permit these muscles to compress the vein during contraction, effectively functioning as alternative venous valves [29]. In the upper face, the angular vein passed through the DS fibres in all specimens; DS contraction can thereby compress the vein and modulate flow from the angular vein into the superior ophthalmic vein and toward the cavernous sinus. In the midface, the vein coursed along the deep surface of the inferior OOc margin in all specimens, where OOc contraction – the largest and most frequently contracting of the relevant muscles – is proposed as the principal modulator of angular vein flow. At the nasal ala, three anatomical course types were identified: between the levator labii superioris (LLS) and Zmi (38.6%), between superficial and deep Zmi fibres (47.7%), or between Zmi and zygomaticus major (13.6%); the first two types allow particularly efficient compression due to the narrow intermuscular space [29]. Experimental evidence corroborates this model: smiling significantly reduces facial vein flow, and forceful OOc contraction decreases angular vein blood flow velocity from approximately 10 cm/s to 7.3 cm/s (Iwanaga et al., 2022). These findings suggest that facial muscle activity constitutes a functional, dynamic valve-equivalent mechanism in the angular vein independent of structural valve leaflets, with implications for the spread of facial infections and for the design of surgical and minimally invasive procedures in this region.

### Retromandibular vein

Valves were observed in a minority of retromandibular veins (5/40 specimens); when found, they were bicuspid with cusps directed towards the heart [32]. Functionally, these valves seem to compartmentalise the vein rather than prevent retrograde flow, consistent with a blood-reservoir function [32].

### Superficial temporal vein

The STV contains both true venous valves and valve-like structures termed venous cristae [67]. Across 24 STVs, 5 bicuspid valves and 69 cristae were identified; valves were in main trunks only, and cristae predominantly at bifurcations. Knowledge of these structures is essential for craniofacial reconstruction because they may cause postoperative venous congestion [67].

### Deep facial vein

The deep facial vein (vena facialis profunda; the “veine faciale profonde” of classical French anatomy) is the principal anastomotic channel connecting the superficial facial venous system to the pterygoid venous plexus (PVP). Paturet (1958) described it as a communicating branch of the facial vein, coursing deep to the masseter muscle and superficial to the buccinator muscle, draining directly into the infratemporal PVP [52]. This classical description has been confirmed in modern cadaveric dissection studies: Siwetz et al. (2023) identified a deep facial vein in 56.3% of 96 hemifaces, with additional parallel tributary vessels present in 37% of those cases [59]. The orifice of the deep facial vein into the facial vein showed no specific pattern regarding its level, reflecting the anatomical variability of this structure. Cotofana et al. (2017) also documented this connection, while Isaac et al. (2025) noted that both the anterior and posterior facial veins communicate with the PVP through such source veins [12, 28].

The deep facial vein is consistently valveless [28, 59], and this constitutes the anatomical substrate for the bypassing of facial vein valves in infection or embolic spread toward the cavernous sinus (see Sect.  9.3, Route C). Its consistent absence of valves also explains the observation by Miyake et al. (1996). In contrast, valves are abundant in the facial vein proper; the communicating pathways to the deeper pterygoid territory lack them entirely. Surgically, inadvertent injury to the deep facial vein during masseteric or infratemporal approaches may cause profuse bleeding due to its direct communication with the PVP, underscoring the clinical relevance of this classically described but frequently underemphasised vessel.

### Glabellar and forehead region

A dedicated cadaveric study of the venous architecture of the glabellar-to-forehead region in 15 fresh specimens identified a consistent communicating vein connecting the bilateral angular veins across the nasal root, termed the transverse nasal root vein [57]. From this vessel, one or two large ascending cutaneous veins drain the forehead dermis via a subdermal polygonal venous network. Three distinct categories of venous valves were identified based on anatomical location: type 1 valves, very small, at the junction between small ascending veins and the polygonal venous network; type 2 valves, small, at the junction between the polygonal venous network and the large ascending veins; and type 3 valves, the largest, at the junction of the large ascending cutaneous veins and the transverse nasal root or angular vein at the medial canthal area [57]. These hierarchically arranged valves prevent reflux from the deep to the superficial venous layer and have direct surgical implications: failure to preserve the main ascending cutaneous vein and its anastomosis with the transverse nasal root vein is a recognised cause of venous congestion and partial flap necrosis in glabellar and forehead reconstructions [57].

## Ophthalmic veins

The history of ophthalmic vein valves reflects the broader trajectory of head and neck valve research: from entrenched scepticism to progressive anatomical recognition. Franklin’s 1927 survey noted that Piersol conceded only ‘occasional’ valves in the angular and ophthalmic veins, while Houzé called them rare and variable in the facial vein. This characterisation substantially understated anatomical reality [19]. An earlier Chinese cadaveric study documented venous valves within the lumen of the superior ophthalmic vein, the infraorbital communicating branch of the angular vein, and the supraorbital vein in 79% of dissected specimens [25]. Zhang and Stringer (2010) subsequently provided a systematic modern demonstration: 10 bicuspid valves in 9/12 (75%) specimens, with cusp orientation directing flow toward the cavernous sinus [70]. Isaac et al. (2024) confirmed this. They extended it to the SOV’s named tributaries, the supraorbital, supratrochlear, and external nasal veins, while finding no valves in the inferior ophthalmic vein (IOV) across 8 specimens examined. The IOV’s consistent absence of valves likely reflects its smaller calibre and distinct haemodynamic environment.

The clinical significance of SOV valve status is illustrated by the carotid-cavernous fistula (CCF). SOV enlargement is a classical imaging hallmark of CCF; however, in a retrospective series of 40 CCF patients, 26% showed no SOV enlargement on non-invasive imaging, and 8% showed none even on catheter angiography, demonstrating that orbital venous drainage can be entirely redirected through petrosal sinuses or the pterygoid plexus without SOV involvement [30]. The orientation of SOV valves toward the cavernous sinus means they facilitate rather than obstruct the forward transmission of fistula congestion; their absence from the drainage pathway or redirection to posterior routes therefore explains presentations without the expected orbital signs [30, 70].

Histopathological evaluation of ophthalmic veins in elderly cadavers (28 subjects; mean age 77.86 ± 13.38 years; 49 orbits evaluated by H&E, Verhoeff, laminin, and CD31 staining) has revealed previously undescribed microscopic features. Vasa vasorum (VV) were identified within OV walls in vessels below 0.5 mm in luminal diameter, and a true valvular structure was documented within one such VV in a 52-year-old male cadaver, extending the evidence for microscopic venous valves to a territory previously regarded as uniformly valveless [41]. Age-related histological changes in the OVs included progressive elastin fragmentation and disorganisation after 66 years, irregular endothelial cell nuclear distribution detectable from 48 years, and collagen fibre disorganisation in elderly subjects – changes that paralleled those described in arteries and large systemic veins. The OV walls share features with superficial cerebral veins of similar calibre, including a thin wall with limited myocyte content, consistent with the common embryological derivation of ophthalmic and intracranial venous structures from the primitive head venous system. These age-related degenerative changes may influence orbital venous haemodynamics and contribute to the elevated prevalence of retinal vein occlusion in elderly individuals [41].

As Zhang and Stringer (2010) concluded, it is not the absence of valves but rather the communication pathways and the direction of cusp orientation that explain the spread of infection from the face to the cavernous sinus via the ophthalmic route (Fig. 3).

## Intracranial veins, dural sinuses, and emissary veins

The intracranial venous system is consistently described as lacking valves and muscular layers [33]. Emissary veins are explicitly valveless, connecting the extracranial scalp to the dural sinuses and diploic veins; this facilitates the spread of infection, air embolism, significant bleeding, and venous thrombosis [33]. Named emissary veins include posterior condylar, mastoid, occipital, and parietal veins; the ophthalmic pathway is better described as an ophthalmic venous route through the superior ophthalmic vein rather than a formally named emissary vein (Figs. 1 and 4).

## Vertebral venous plexuses: valvular architecture and haemodynamic implications

The vertebral venous system, commonly referred to as Batson’s plexus, exhibits a structurally significant valvular dichotomy between its internal and external components. The internal vertebral venous plexus (IVVP), occupying the epidural space, is characteristically avalvular, with a muscular, trabecular wall that permits bidirectional, pressure-dependent flow throughout the craniospinal axis [4, 60]. The external vertebral venous plexus (EVVP), by contrast, is equipped with valves that orient flow preferentially from the external paravertebral and posterior muscular venous networks inward through intervertebral/radicular communications toward the epidural IVVP, providing directional regulation at the interface between the two compartments [36, 60]. The vertebral veins proper, draining via the transverse foraminal plexuses into the brachiocephalic vein, also carry valves, though their disposition exhibits anatomical variability [39, 43].

This asymmetry, valved external tributaries converging on a valveless internal plexus, underlies the system’s capacity to function as a low-pressure, high-capacity collateral route when caval return is compromised, while simultaneously enabling retrograde propagation of emboli, infection, and neoplastic cells along the craniospinal axis [7, 9, 46]. The absence of valves within the IVVP, combined with its direct communication with the dural venous sinuses at the craniocervical junction [65], positions this plexus as a critical and largely unguarded conduit between intracranial and extracranial venous territories, a consideration of direct relevance to the haemodynamic scope of the present review.

## Lymphovenous valve at the thoracic duct–venous junction

On the left side of the neck, the thoracic duct (TD) empties into the venous system at the lymphovenous junction (LVJ), a site guarded in most, but not all, individuals by a specialised lymphovenous valve (LVV) (Table 1). In a cadaveric series of 21 LVJs, a discrete valve was identified in approximately 71% of specimens, with the remaining 29% lacking any valvular structure [51]. When present, the LVV is most commonly bicuspid and semilunar in configuration; histologically, the cusps consist of endothelial folds supported by a thin collagen core, with ostial and flap-like variants also documented [20, 50, 51].

**Table 1:** Morphology and variability of the lymphovenous valve

Feature	Typical findings	References
Prevalence	Present in ~ 71% of LVJs	[51]
Morphology	Bicuspid semilunar; ostial/flap-like variants	[20, 51]
Histology	Endothelial folds with thin collagen core	[51]
Absence	No discrete valve in ~ 29% of junctions	[51]

The LVV operates in concert with respiratory-driven pressure fluctuations to permit unidirectional lymph entry into the venous system. During inspiration, falling central venous pressure (CVP) allows the valve cusps to collapse, facilitating lymph outflow; during early expiration, rising CVP closes the cusps and limits reflux [16, 53]. This dynamic behaviour is supported by ultrasound evidence demonstrating that terminal TD diameter varies measurably with both respiratory phase and body position [24]. Chronic CVP elevation, as in heart failure or cirrhosis, impairs TD drainage despite increased lymph production, underscoring the haemodynamic dependency of LVJ patency on downstream venous pressure [53].

Where the valve is structurally weak or absent, additional mechanisms sustain blood–lymph separation. Platelet-mediated thrombi at the LVJ have been shown in murine models to maintain separation throughout life [23], and the tangential, intramural course of the TD through the venous wall may itself reduce retrograde blood entry into the lymphatic lumen [20, 50]. The frequent absence of a discrete LVV therefore does not necessarily denote an unprotected junction, but rather reflects a multimodal system in which valvular, haemostatic, and architectural elements operate in concert.

## Clinical significance

### Central venous access and cerebral hemodynamics

The brachiocephalic vein and SVC lack valves, enabling right atrial contraction to transmit flow directly to the inferior bulb of the IJV. The IJV valve is the sole valve between the right atrium and the brain [27, 64] and plays a critical role in maintaining the extrathoracic arteriovenous pressure gradient during cardiopulmonary resuscitation and in shielding the cerebral venous system from acute rises in intrathoracic pressure [18]. Variability and frequent incompetence of the IJV valve may influence cerebral venous haemodynamics by permitting intermittent retrograde transmission of central venous pressure, particularly during Valsalva manoeuvres, coughing, positive-pressure ventilation, or conditions associated with chronically elevated central venous pressure.

### Microsurgical reconstruction

Valve mapping in the EJV, facial vein, and STV is important for head and neck reconstruction because valves can hinder outflow and lead to postoperative congestion or thrombosis [1, 15, 47, 48, 67]. Surgical strategies involve resecting about twice the inner diameter at the planned anastomosis sites [47, 48].

### Infection spread and intracranial pathology

Multiple valveless pathways connect the face to the cavernous sinus (Fig. 3). Route A (via SOV) illustrates that valves do not prevent spread, as SOV cusps direct flow towards the sinus [70]. Route B proceeds through valveless emissary veins [33] (Fig. 4). Route C bypasses facial vein valves via the valveless deep facial vein (DFV) and pterygoid plexus: the DFV, classically described by Paturet (1958) as a communicating branch linking the facial vein to the infratemporal pterygoid venous plexus (PVP), is present in approximately 56% of individuals [59] and provides a consistently valveless alternative route that circumvents any protective effect of facial vein valves. Additionally, a recently described anatomic variant, the parapharyngeal vein, provides a previously unrecognised direct accessory drainage pathway from the superficial middle cerebral vein (SMCV) via the pterygoid plexus directly into the IJV, bypassing the usual retromandibular and facial vein route entirely [54]. This vein, which courses through the parapharyngeal space between the medial pterygoid muscle and the pharyngeal wall anteromedially to the internal carotid artery, may channel venous outflow through a single pressure-sensitive route, and its inadvertent injury during neck dissection or skull base surgery carries a risk of significant bleeding or postoperative infarction in the absence of alternative drainage [54]. The current view suggests that it is not the absence of valves but rather communication pathways and flow direction that account for the spread of intracranial infection [28, 43, 70].

### Metastatic spread

The valveless IVVP (Batson’s plexus) offers a recognised pathway for metastatic spread from the pelvis to the vertebral column and brain, bypassing the pulmonary filter [8].

### Temporal augmentation and venous embolic risk

Temporal-region venous anatomy also has implications for aesthetic procedures. In a cadaveric study of the middle temporal vein (MTV), Tansatit et al. demonstrated a direct anterograde channel from the MTV to the internal jugular vein. They found that retrograde filler passage towards the orbital venous tributaries was difficult in their model. These findings suggest that inadvertent cannulation of the MTV during temporal augmentation may favour pulmonary venous embolic complications rather than ocular complications. The authors further reported that compression of the pretragal/preauricular venous confluent point retarded anterograde flow, indicating a possible preventive manoeuvre, although this did not eliminate risk [62].

## Limitations

As a narrative review, this synthesis is limited by the heterogeneous nature of the available evidence. Several key observations derive from cadaveric studies, corrosion casts, ultrasound cohorts, or small anatomical series that use different definitions of valve presence and competence. Functional extrapolation from anatomic valve morphology to clinical protection must therefore be cautious, particularly for cerebral venous haemodynamics, infection spread, and microsurgical outcomes. The available literature also varies in specimen age, laterality reporting, imaging protocols, manoeuvres used to provoke reflux, and completeness of named venous pathway descriptions.

## Summary

Table 2 summarises valve status across selected head and neck venous structures.

**Table 2:** Valve status in the head and neck veins

Structure	Valve status	Key details and references
IJV	Present; variable	93% cadaveric [37]; 86% US/240 IJVs [38]; 88% US/462 IJVs [66]; absent ∼12–13% [2,22]; bicuspid predominant (66% cadaveric; 75% US); right–left asymmetry [56]; 100% present in cadavers; competent to median 56.8 mmHg in vitro and in all live subjects during 60 mmHg Valsalva [58]; 95% incompetent on insufflation [38]; 90% incompetent by colour Doppler, competent valves only in those < 20 yrs [66]; higher competence in children, declining with age [13]; iatrogenic incompetence from cannulation [68]
EJV	Present	Terminal + mid-portion [48]; terminal valve prevents subclavian regurgitation [33] ; extensive morphological variability, including fenestrations, duplications, and absence [55]
AJV	Valveless	Infection pathway to the cavernous sinus [3]
Facial vein	Present in 82%	82% at confluences; 93.5% bicuspid [47]; ostial/parietal types [45]; SEM reveals valves to < 150–200 μm diameter; leaflets ∼4–6 μm, exclusively bicuspid; well-formed in motile regions [44]; 17 valves in angular/facial tributaries, all bicuspid [70]
Angular vein	No leaflet valves; muscular compression	Courses through/between DS, OOc, and Zmi in all 44 specimens; muscle contraction compresses the vein as a functional valve equivalent; 3 anatomical types at the nasal ala; OOc contraction reduces flow from 10 to 7.3 cm/s [29]
Retromandibular V.	Minority (12.5%)	5/40 veins; bicuspid; compartmentalising function [32]
STV	Valves + cristae	5 bicuspid valves + 69 cristae / 24 STVs; valves in trunks, cristae at bifurcations [67]
Glabellar/forehead veins	Present (3 types)	Three hierarchical valve types at branching sites of ascending cutaneous veins; the transverse nasal root vein is consistently present, connecting bilateral angular veins; valve preservation is critical for flap survival [57]
SOV	Present in 75%	Valves in 79% specimens [25]; 10 valves / 9 of 12 specimens, all bicuspid, cusps directing flow toward cavernous sinus [70]; confirmed in SOV tributaries [28]; microscopic valve in VV of OV [41]; SOV may not enlarge in CCF if drainage diverted posteriorly [30]
IOV	Absent	0/8 specimens [70]
Emissary veins	Valveless	Bidirectional; infection/air embolism risk [6,11,33,40]
Cerebral vv. / dural sinuses	Valveless	No muscular walls or valves [33]
IVVP	Valveless	Batson’s plexus; metastatic spread to the pelvis → brain [8]
Lymphovenous junction	Present in 71%	Bicuspid; thoracic duct–venous junction [51]

Contemporary evidence indicates that many head and neck veins contain valves, particularly the facial, lingual, labial, pharyngeal, SOV, STV, and IJV (Fig. 1). These valves are predominantly bicuspid and cluster near junctions. The traditional textbook characterisation of a uniformly valveless head and neck venous system requires revision.

## Data Availability

No datasets were generated or analysed during the current study.

## Abbreviations

AJV: Anterior jugular vein
CPR: Cardiopulmonary resuscitation
CST: Cavernous sinus thrombosis
EJV: External jugular vein
EVVP: External vertebral venous plexus
IIH: Idiopathic intracranial hypertension
IJV: Internal jugular vein
IOV: Inferior ophthalmic vein
IVVP: Internal vertebral venous plexus
LVJ: Lymphovenous junction
LVV: Lymphovenous valve
SANRA: Scale for the assessment of narrative review articles
SOV: Superior ophthalmic vein
STV: Superficial temporal vein
SVC: Superior vena cava
DFV: Deep facial vein
PVP: Pterygoid venous plexus
TD: Thoracic duct
